# Nisin a probiotic bacteriocin mitigates brain microbiome dysbiosis and Alzheimer’s disease-like neuroinflammation triggered by periodontal disease

**DOI:** 10.1186/s12974-023-02915-6

**Published:** 2023-10-06

**Authors:** Chuanjiang Zhao, Ryutaro Kuraji, Changchang Ye, Li Gao, Allan Radaic, Pachiyappan Kamarajan, Yoshimasa Taketani, Yvonne L. Kapila

**Affiliations:** 1 Department of Orofacial Sciences, School of Dentistry, University of California San Francisco, San Francisco, CA 94143 USA; 2 Department of Biosystems and Function and Periodontics, School of Dentistry, University of California Los Angeles, Los Angeles, CA 90024 USA; 3 Department of Periodontology, Hospital of Stomatology, Sun Yat-Sen University, Guangzhou, 510050 China; 4 Guangdong Provincial Key Laboratory of Stomatology, Guanghua School of Stomatology, Sun Yat-Sen University, Guangzhou, 510050 China; 5 Department of Periodontology, The Nippon Dental University School of Life Dentistry at Tokyo, Tokyo, 102-8159 Japan; 6 Department of Periodontology, West China School of Stomatology, National Clinical Research Center for Oral Diseases, State Key Laboratory of Oral Diseases, Sichuan University, Chengdu, 610093 China; 7 Division of Periodontology, Department of Oral Biology and Tissue Engineering, Meikai University School of Dentistry, Sakado, 350-0283 Japan; 8 Section of Biosystems and Function, Section of Periodontology, UCLA School of Dentistry, 10833 Le Conte Ave, Box 951668, Los Angeles, CA 90095-1668 USA

**Keywords:** Oral microbiome, Brain microbiome, Periodontal disease, Neuroinflammation, Nisin, Antimicrobial therapy

## Abstract

**Introduction:**

Periodontitis-related oral microbial dysbiosis is thought to contribute to Alzheimer's disease (AD) neuroinflammation and brain amyloid production. Since probiotics can modulate periodontitis/oral dysbiosis, this study examined the effects of a probiotic/lantibiotic, nisin, in modulating brain pathology triggered by periodontitis.

**Methods:**

A polymicrobial mouse model of periodontal disease was used to evaluate the effects of this disease on brain microbiome dysbiosis, neuroinflammation, Alzheimer’s-related changes, and nisin’s therapeutic potential in this context.

**Results:**

16S sequencing and real-time PCR data revealed that Nisin treatment mitigated the changes in the brain microbiome composition, diversity, and community structure, and reduced the levels of periodontal pathogen DNA in the brain induced by periodontal disease. Nisin treatment significantly decreased the mRNA expression of pro-inflammatory cytokines (Interleukin-1β/IL-1 β, Interleukin 6/IL-6, and Tumor Necrosis Factor α/TNF-α) in the brain that were elevated by periodontal infection. In addition, the concentrations of amyloid-β 42 (Aβ42), total Tau, and Tau (pS199) (445.69 ± 120.03, 1420.85 ± 331.40, 137.20 ± 36.01) were significantly higher in the infection group compared to the control group (193.01 ± 31.82, 384.27 ± 363.93, 6.09 ± 10.85), respectively. Nisin treatment markedly reduced the Aβ42 (261.80 ± 52.50), total Tau (865.37 ± 304.93), and phosphorylated Tau (82.53 ± 15.77) deposition in the brain of the infection group.

**Discussion:**

Nisin abrogation of brain microbiome dysbiosis induces beneficial effects on AD-like pathogenic changes and neuroinflammation, and thereby may serve as a potential therapeutic for periodontal–dysbiosis-related AD.

**Supplementary Information:**

The online version contains supplementary material available at 10.1186/s12974-023-02915-6.

## Background

Periodontitis, a chronic inflammatory disease triggered by an oral microbial dysbiosis in a susceptible host, is one of the most prevalent diseases affecting nearly 50% of the population worldwide [1]. In periodontitis, periodontal tissues are infected by oral microorganisms resulting in destruction of tooth supporting tissues and eventually tooth loss [2]. In addition, evidence has accumulated that links periodontal inflammation with many systemic diseases, including diabetes, cardiovascular disease, cancer, adverse pregnancy outcomes, and neurodegenerative diseases [3–5]. Although the association between periodontal disease and systemic diseases is well-known, the underlying mechanisms are not fully understood. Evidence indicates that the inflammatory response induced by periodontal disease is not confined to periodontal tissues [6]. Instead, oral microorganisms and inflammatory mediators can affect other organs through their circulation in the bloodstream via bacteremias [7]. Levels of IL-1β, IL-6, IL-17, TNF-α, and systemic C-reactive protein are elevated in patients with periodontal disease [8–10]. Ultimately, the synergistic effects of the bacterial infection and immune response originating from the periodontal tissues may contribute to various systemic diseases. Therefore, control of periodontal infections is considered an important strategy for the prevention and treatment of a series of systemic conditions [11–13].

Alzheimer’s disease (AD), the most common form of dementia, is the leading cause of cognitive disorders [14]. AD is a complex, multifactorial disease affecting about 50 million individuals globally. The characteristic pathological changes of AD brains are the accumulation of intracellular hyperphosphorylated tau-positive neurofibrillary tangles (NFT) and insoluble amyloid β(Aβ) plaques, which stimulate glial cell activation and elicit local innate immune responses [15]. The main etiologic factors of AD include brain hypoperfusion, traumatic brain injury, autoimmune disorders, insulin resistance, and other infectious diseases leading to neuroinflammation [16]. Recently, considerable progress has been made in understanding the pathogenesis, diagnosis, and treatment of AD. However, there are no effective therapies to prevent or treat the condition until now [17]. Prevention via modifiable factors is a promising avenue for slowing down the progression of this disease [18, 19].

Growing evidence from cross sectional and longitudinal studies have demonstrated an association between AD and infectious conditions, such as periodontal disease [20]. The presence of periodontal pathogens, such as *Porphyromonas gingivalis* and *Treponema species,* have been found in post-mortem brain tissues of AD patients [21, 22]. In addition, animal studies have been conducted to determine the effects of periodontitis on pathogen translocation and possible effects on the brain. Periodontal pathogens and their molecules have also been reported in the brain [23, 24]. Overall, these findings strengthen the hypothesis that periodontal pathogens play an important role in the development of AD. Periodontal disease and its characteristic oral microbial dysbiosis are thought to contribute to neuroinflammation and amyloid protein production via translocation of periodontal pathogens and their components to the brain. This hypothesis is further supported by research linking the presence of periodontal microorganisms with increased Amyloid β deposition, tau hyperphosphorylation, and glial cell inflammation and activation [25–27]. A recent study showed that treatment with a gingipain inhibitor reduced *P. gingivalis* infection in the brain of mice and prevented further neurodegeneration and accumulation of pathologic plaques, suggesting that controlling periodontal infections and microorganisms may be an effective way to slow down the development of AD [28].

Despite this compelling evidence for the presence of bacteria in the brain supporting the concept of a brain microbiome, there is considerable debate that a brain microbiome exists in healthy individuals [29]. Currently the evidence for the infection of the brain in mouse studies is based on detection of bacterial products, including DNA, RNA, LPS and surface proteins, yet microbiome sequencing studies have not been performed. Here, we examine the presence of a brain microbiome using a genomic method-driven approach [30].

Nisin, a class I Lantibiotic bacteriocin produced by the probiotic *Lactococcus lactis*, has shown efficacy in treating a variety of infectious diseases, including gastrointestinal infections, respiratory tract infections, skin/soft tissue infections, and oral infectious diseases, including periodontal disease [31]. In addition, we’ve demonstrated that nisin treatment can decrease the levels of gram negative periodontal pathogens in planktonic form and in oral biofilms and return their microbial diversity back to control ‘healthy’ levels [31–33]. Nisin can also prevent periodontal disease-related bone loss and inflammation while promoting reparative proliferation and a healthy microbiome [34]. Therefore, the objective of this study was to examine the potential role of nisin in modulating brain microbiome dysbiosis (using sequencing methods), neuroinflammation, and amyloid-β and tau production after polymicrobial periodontal disease.

## Methods

### Polymicrobial infection and treatment of mice

A total of 24 eight-week-old BALB/cByJ female mice (The Jackson Laboratories, Bar Harbor, ME, USA) were housed with enrichment in microisolator plastic cages and randomly distributed into 4 groups (6 mice per group). Mice were monitored daily. The experimental procedures were approved by the Institutional Animal Care and Use Committee of the University of California, San Francisco (IACUC approval number: AN171564-01B). All the mice were given trimethoprim (0.17 mg per ml) and sulfamethoxazole (0.87 mg per ml) daily for 7 days in the drinking water and their oral cavity was rinsed with 0.12% chlorhexidine gluconate (Peridex) mouth rinse to inhibit the native oral microbiota as described previously [35]. The polymicrobial inoculum (5 × 10^9^ combined bacteria per ml; 1 × 10^9^ cells in 0.2 ml per mouse; 2.5 × 10^8^ *P. gingivalis*, 2.5 × 10^8^ T*. denticola*, 2.5 × 10^8^ T*. forsythia* and 2.5 × 10^8^ *F. nucleatum*) was prepared in a 4% (w/v) carboxymethyl cellulose (CMC) solution and administered topically in the morning for 4 consecutive days every week for a total of 8 weeks as described previously [35]. Nisin (300 μg/ml, 0.2 ml per mouse) was administered every day in the evening every week for a total of 8 weeks. A sterile 4% (w/v) carboxymethyl cellulose solution was administered as the control treatment. The polymicrobial inoculum and nisin treatment were administered in alternating fashion (AM and PM) to minimize the stress on the animals. At 8 weeks following the polymicrobial infection, the mice were anaesthetized by CO_2_ inhalation and sacrificed using cervical dislocation. After the mice were sacrificed, the fresh brain tissues without perfusion were immediately frozen in liquid nitrogen and stored frozen until used for subsequent assays.

### Periodontal bacteria and polymicrobial inoculum

The following periodontal pathogens, namely, *P. gingivalis* FDC 381, *T. denticola* ATCC 35405, *T. forsythia* ATCC 43037, and *F. nucleatum* ATCC 10953, were obtained from ATCC (Manassus, VA, USA) and cultured anaerobically (85% N_2_, 10% H_2_, 5% CO_2_) at 37 °C according to methods described in our previous study [35]. *P. gingivalis and F. nucleatum* were grown for 3 days in Tryptic Soy Broth (Becton Dickinson, Franklin Lakes, NJ) supplemented with 5 mg/ml yeast extract, 0.5 mg/ml l-cysteine hydrochloride, 5 μg/ml hemin, 1 μg/ml menadione and 5% fetal bovine serum (FBS) (Gibco Thermo Fisher Scientific, Waltham, MA). *T. denticola* was cultured in Oral Treponeme Enrichment Broth medium (Anaerobe systems, Morgan Hill, CA, USA) for 5 days. *T. forsythia* was grown for 7 days in Tryptic Soy Broth containing 5 mg/ml yeast extract, 0.5 mg/ml l-cysteine hydrochloride, 5 μg/ml hemin, 1 μg/ml menadione, 10 μg/ml *N*-acetylmuramic acid (Sigma-Aldrich, St. Louis, MO, USA), and 5% FBS. The cell density was determined quantitatively using a spectrophotometer and each organism was resuspended in phosphate-buffered saline (PBS) at 1 × 10^10^ bacteria per ml for experiments.

For the oral polymicrobial infection, *P. gingivalis* was mixed with an equal volume of *T. denticola* for 5 min. Subsequently, *T. forsythia* was added to the culture tubes containing *P. gingivalis* and *T. denticola*, and the bacteria were mixed gently for 1 min and allowed to interact for an additional 5 min. Finally, *F. nucleatum* was added and mixed well with *P. gingivalis*, *T. denticola*, and *T. forsythia.* After 5 min, the four bacterial consortia were mixed thoroughly with an equal volume of sterile 4% (w/v) CMC in PBS, and this mixture was used as the polymicrobial oral inoculum.

Furthermore, we quantified the concentration of all four bacteria, ensuring a concentration of 2.5 × 10^8^ four periodontal pathogens (*P. gingivalis, T. denticola, T. forsythia*, and *F. nucleatum*) and the purity and viability of the four bacteria were confirmed by microscopic evaluation and colony morphology prior to inoculation. Finally, we’ve shown in this very mouse model at the end of the experimental period after euthanasia that these bacteria are all present locally in the gingiva of the mice and that they mount a systemic antibody response to each pathogen, indicating the viability of each pathogen in the inoculation step [34]. Although, we have found that it is relatively hard for *T. denticola* to colonize the oral cavity compared with the other three bacteria, we have cultured *T. denticola* strictly according to our typical protocols (3234-40), and third and fifth passage *T. denticola* preparations were used in this experiment.

### Nisin preparation

An ultra-pure (> 95%) food grade form of nisin Z (NisinZ^®^ P) was purchased from Handary (S.A., Brussels, Belgium), a primary manufacturer of nisin in the food industry. The nisin stock solution was prepared at a concentration of 600 μg/ml in sterile Milli-Q filtered water, that was further filtered using a 0.22 μm syringe filter, and stored at 4 ℃ for a maximum of 5 days for use in experiments [33, 34]. For oral treatment of mice, the nisin solution was then mixed with an equal volume of sterile 4% CMC and adjusted to the final concentration of 300 μg/ml.

### DNA isolation from brain tissues

DNA was extracted from the mouse brain tissues to evaluate microbiological changes following periodontal bacterial challenge and/or nisin treatment using real-time polymerase chain reaction (PCR) and 16s rRNA sequencing. DNA was isolated and purified using the QIAamp^®^ DNA Mini kit (Qiagen, Hilden, Germany) as in our previous study [34, 35]. The isolated DNA was stored at − 20 °C until further processing for real-time PCR and 16s rRNA sequencing analysis.

### RNA isolation from brain tissues

For RNA stabilization, the mouse brain tissues were treated immediately after sample collection with an RNAlater solution (Invitrogen) at 4 ºC overnight. Samples were powdered with a mortar and pestle under continuous liquid nitrogen, and total RNA was then isolated from each sample using an RNeasy Lipid Tissue Mini Kit (QIAGEN). The purity and quantity of the RNA were evaluated using the NanoVue Plus spectrophotometer (Biochrom Ltd.). Subsequently, total RNA was synthesized into cDNA using a High-Capacity cDNA Reverse Transcription Kit (Applied Biosystems) and according to manufacturer’s protocols.

### Bacterial isolation from brain tissues

Using a slightly different mouse model from the model described above, with the only difference being that these mice were orally inoculated with *T. denticola* (ATCC 35405), fresh brain specimens from these mice were sampled with swabs to attempt to grow out whole living bacterial microbes using a sterile technique/environment. For this purpose, brain samples were dissected and cut in half in a sagittal plan, then swab samples were collected from the inner area of the left and right halves of the brain and the whole brain. These swab samples were grown on Oral Treponeme Enrichment Broth media anaerobically at 37 ºC for 72 h. Subsequently, turbid media, indicating bacterial growth, was sampled and checked by PCR for bacterial growth of 4 periodontal pathogens [*T. denticola, P. gingivalis, F. nucleaturm, T. forsythia* as measured by PCR using TaqMan primers and probes (Invitrogen) corresponding to the 16S rRNA gene as in our previous study [34, 35]]; revealing the detection of *F. nucleatum* in the samples from the left half of the brain. Subsequently the samples from the left half of the brain were plated on BHI agar plates (Anaerobe systems, CA) for 72 h. Individual colonies were randomly selected from the plates and expanded for another 72 h in BHI media. DNA was isolated from these bacterial broth samples and checked for the presence of the four specific periodontal pathogens (*T. denticola, P. gingivalis, F. nucleaturm, T. forsythia*).

### Microbiome analysis of brain tissues via 16s rRNA sequencing

The purity and quantity of DNA samples isolated from brain tissues were deemed suitable and met quality control measures for 16s rRNA sequence performed by Novogene, Inc. (en.novogene.com). For the sequencing library preparation, the V4 variable region (515F-806R) of the samples was amplified using specific barcoded primers. All PCR reactions were carried out with Phusion^®^ High-Fidelity PCR Master Mix (New England Biolabs) and the PCR products were purified with Qiagen Gel Extraction Kit (Qiagen, Germany). The libraries were generated with the NEBNext^®^ UltraTM DNA Library Prep Kit for Illumina and were then sequenced using the Illumina NovaSeq 6000 System.

### Quantification of periodontal pathogens by real-time PCR

Quantification by standard real-time PCR was used to evaluate the abundance of the periodontal pathogens in the brain tissue samples. Four periodontal pathogens used for the polymicrobial infection were measured by PCR using TaqMan primers and probes (Invitrogen) corresponding to the 16S rRNA gene as in our previous study [34, 35]. To this end, tenfold serial dilutions of known concentrations of the DNA of the periodontal pathogens were used to construct standard curves for quantification of the periodontal pathogens in the samples. Next, 100 ng of extracted sample DNA was processed for real-time PCR analysis to obtain the crossing point (Cp) value, then we converted the copy number of the bacteria based on the standard curve and Cp value to evaluate the abundance of the periodontal pathogens in the brain tissue samples. The amplification was conducted using a QuantStudio 3 Real-Time PCR system (Thermo Fisher Scientific) with a final reaction volume of 20 μl, including TaqMan Fast Advanced Master Mix (Applied Biosystems), DNA (15 ng/μl), primers, and probes. The optimized thermal cycling conditions were as follows: 95 °C for 10 min followed by 50 cycles of denaturing at 95 °C for 15 s, annealing and extension at 60 °C for 1 min. Data were analyzed using QuantStudioTM Design and Analysis Software v1.4.3 (Thermo).

### Real-time PCR evaluation of gene expression from brain tissues

To evaluate the immune cytokine profiles from brain tissues, relative gene expression was measured by real-time PCR using the following TaqMan primers and probes (TaqMan Gene Expression Assays; Applied Biosystems): interleukin 1β (*Il1β*; Mm00434228_m1), *IL-6* (*Il6*; Mm00446190_m1), tumor necrosis factor-α (*Tnf*; Mm00443258_m1). Glyceraldehyde 3-phosphate dehydrogenase (*Gapdh*; Mm99999915_g1) was used as a housekeeping gene to normalize the amount of mRNA present in each reaction. PCR was performed in 20 μl reaction mixtures containing the TaqMan Fast Advanced Master Mix, cDNA template (20 ng/μl well), primers, and probes. The optimized thermal cycling conditions were as follows: 20 min at 95 °C, followed by 40 cycles per 1 min at 95 °C, and 20 min at 60 °C. To compare the expression levels among different samples, the relative expression level of the genes was calculated by the comparative cycle threshold (ΔΔCT) method using QuantStudioTM Design and Analysis Software.

### Determination of the 42 amino acid form of amyloid-b (Aβ42), total Tau, and phosphorylated Tau (pS199) in brain tissues by enzyme-linked immuno-sorbent assay (ELISA)

Brain tissues were harvested and frozen at − 80 ℃ until ready for use. Aβ42, total tau and phosphorylated tau (pS199) levels were assessed utilizing a commercially available ELISA kit (Invitrogen, Aβ42: #KMB3441, total tau: #KMB7011, pS199: #KMB7041). Briefly, brain tissue was homogenized in eight volumes of 5 M guanidine–HCL/50 mM Tris–HCL at pH 8.0, and then mixed and incubated for 3 h at room temperature. After the incubation, the homogenates were dissolved in 1 × protease inhibitor (Sigma) diluted tenfold times with cold PBS, and subsequently centrifuged at 16,000×*g* for 20 min at 4 °C. The supernatant was transferred to new tubes and further diluted with standard diluent buffer. Afterwards, levels of Aβ42, tau and phosphorylated tau (pS199) in the samples were quantitatively assessed by a sandwich ELISA as per the manufacturer’s directions. Data were expressed in pg/mL of homogenate.

### Statistical analysis

SPSS 21.0 statistical software (IBM, Chicago, IL, USA) was used for the statistical analyses. The comparison of bacterial numbers, immune profile-related gene expression, and Aβ42, tau and tau (pS199) levels were analyzed using ANOVA and Tukey’s test for multiple comparison among 4 groups. ANOVA and Tukey’s test for multiple comparison were also used for analysis of α-diversity indices, including Chao1, Shannon and Simpson index. In addition, *t* test was performed to evaluate the Analysis of Similarity (ANOSIM) between two groups. A *P* value less than 0.05 was considered to be significant. A sample of 6 mice per group was selected based on earlier studies with this polymicrobial mouse model of periodontal disease indicating that this sample size yielded sufficient power to detect changes in various oral parameters and microbiome changes [34, 35, 41].

## Results

### Oral polymicrobial infection/periodontal disease shifts the brain microbiome composition and nisin reverses the change

**Fig. 1:**
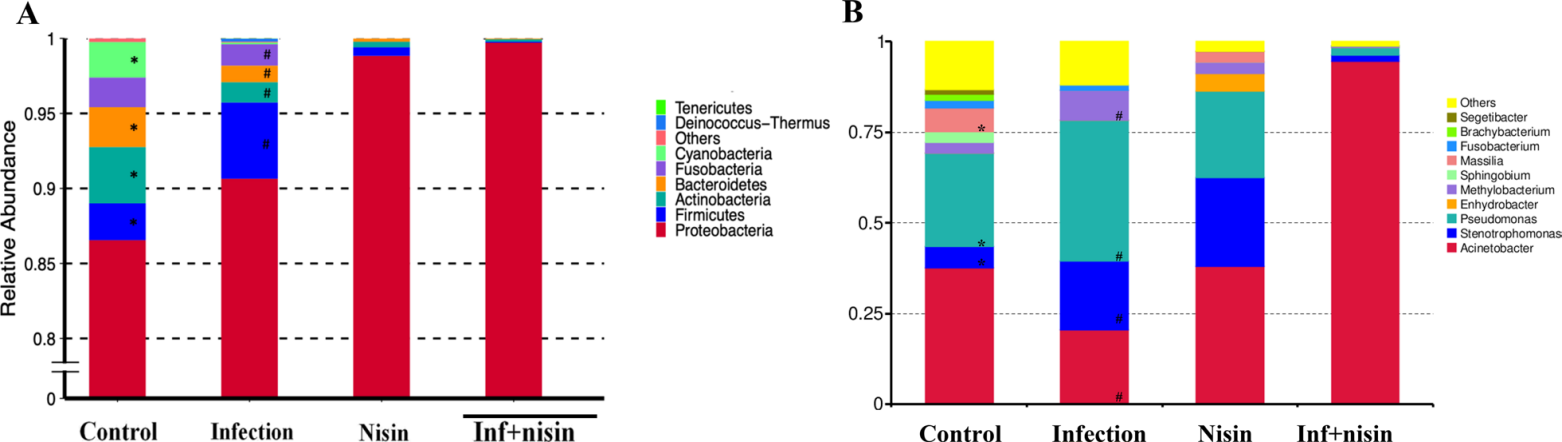
Analysis of the microbial abundance by 16s rRNA sequencing shows that nisin reverses the changes in brain microbiome composition induced by oral polymicrobial infection. The groups included Control, Infection, Nisin, Infection + Nisin. Differential abundance analysis for bacteria at phylum (**A**) and genus level (**B**). *, the difference between the Control and Infection group was significant (*P* < 0.05). #, the difference between the Infection and Infection + Nisin group was significant (*P* < 0.05)

To assess effects of the periodontal polymicrobial infection/periodontal disease and nisin treatment on the brain microbiome, the microbial composition and abundance were analyzed by 16s rRNA sequencing and Metastat analysis at the phylum and genus level. At the phylum level, the relative abundance of *Firmicutes* and *Proteobacteria* were higher in the infected mice than in the healthy control mice, whereas the proportion of *Actinobacteria*, *Bacteroidetes* and *Cyanobacteria* were lower; although *Proteobacteria* did not reach statistical significance (Fig. 1A). In contrast, nisin treatment alone or in the context of infection shifted the microbial composition by increasing the relative abundance of *Proteobacteria* but decreasing the proportion of *Firmicutes*, *Actinobacteria*, *Bacteroidetes* and *Fusobacteria* (Fig. 1A). At the genus level, the proportion of *Stenotrophomonas* and *Pseudomonas* significantly increased in the infection group compared to the control group (*P* < 0.05), whereas the relative abundance of *Acinetobacter*, *Sphingobium*, *Massilia*, *Branchybacterium* and *Segetibacter* decreased (Fig. 1B). Furthermore, the administration of nisin recovered the disease-associated changes by significantly increasing the abundance of *Acinetobacter* (*P* < 0.05), and reducing the proportion of *Stenotrophomonas*, *Pseudomonas* and *Methylobacterium* (*P* < 0.05) (Fig. 1B). Nisin similarly abrogated the oral microbial dysbiosis triggered by the periodontal disease, as previously reported [34, 41].

### Oral polymicrobial infection/periodontal disease shifts the brain microbiome diversity and community structure, and nisin reverses the change

**Fig. 2:**
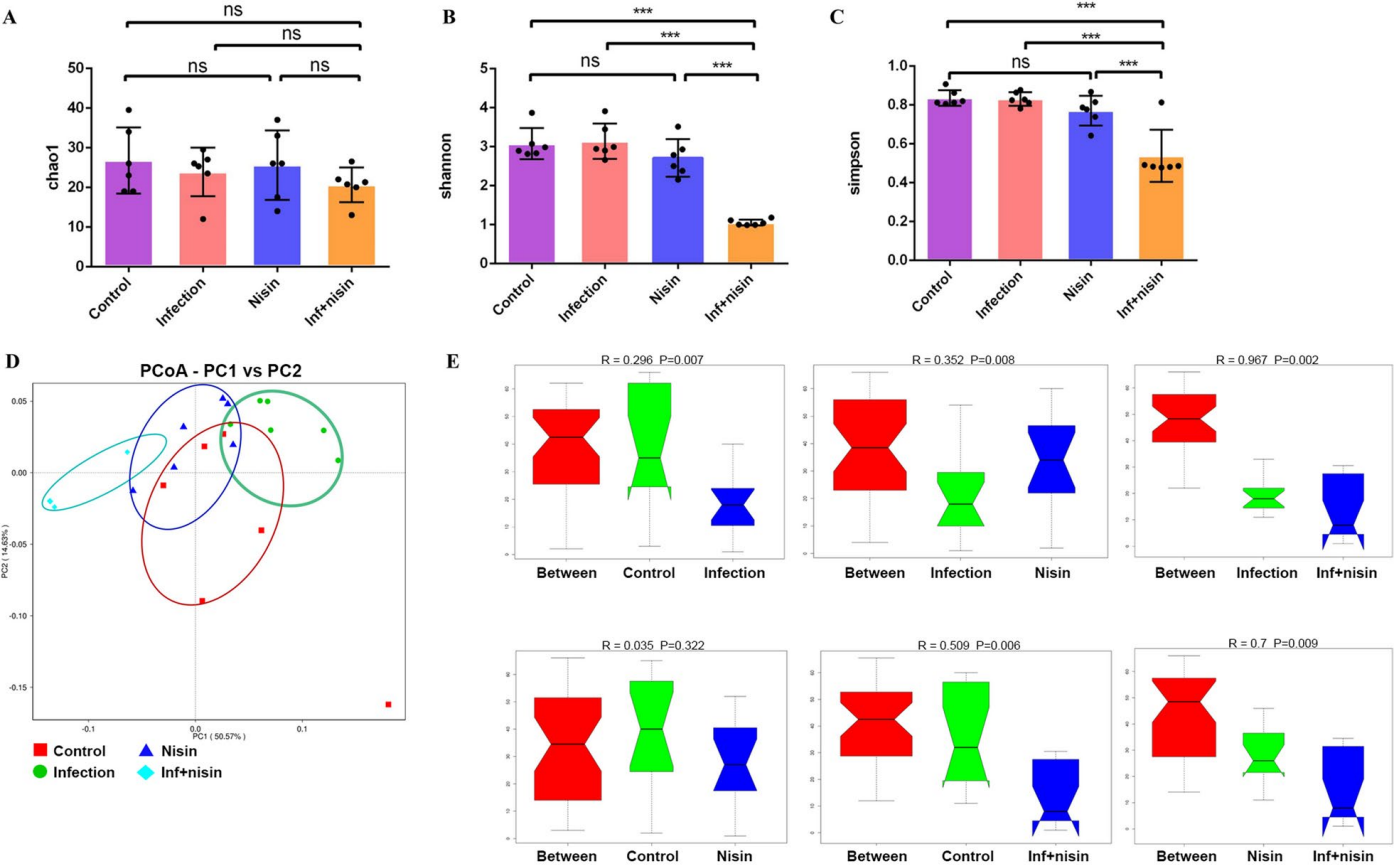
Analysis of microbial community composition and diversity shows that nisin alters microbial diversity and community structure in brain following oral polymicrobial infection. The groups included Control, Infection, Nisin, Infection + Nisin. **A**–**C** Chao1 estimator, Shannon index and Simpson index are analyzed based on the numbers of OTUs from brain tissues. There is no significant difference in Chao1 among the four groups. As for Shannon index and Simpson index, the bacterial diversity score of the Infection + Nisin group is significantly lower than that of the Control, Infection and Nisin group. **D** PCoA based on weighted Unifrac distance is shown for different groups. The microbial compositions of Infection and Infection + Nisin group are shifting to different states, while the microbial compositions of Control and Nisin group are in the middle state. **E** Analysis of Similarity (Anosim) among different groups are shown. The microbiome compositions of the Control, Nisin and Infection + Nisin group are significantly different from that of the Infection group

To assess the changes in brain bacterial diversity following oral polymicrobial infection and nisin treatment, the Chao1 estimator, Shannon index and Simpson index were analyzed based on the numbers of OTUs in the brain tissues. As shown in Fig. 2A, there was no significant difference in the community richness among the four groups (*P* > 0.05). As for the Shannon and Simpson indices, the bacterial diversity score of the infection + nisin group was significantly lower than that of the control, infection and nisin group (*P* < 0.001) (Fig. 2B, C).

To evaluate the overall changes in the microbiome, we further performed Principal Coordinate Analysis (PCoA) based on the Weighted Unifrac distance and analysis of similarities (ANOSIM) (Fig. 2D, E). Interestingly, we found that the brain microbiome of the control and nisin group was in the middle state between the infection and infection + nisin group (Fig. 2D). The microbiome composition of the control, nisin, and infection + nisin group were significantly different from that of the infection group (*P* = 0.007 with *R* = 0.296, *P* = 0.008 with *R* = 0.352 and *P* = 0.002 with *R* = 0.967, respectively) (Fig. 2E). In addition, the microbiome composition of the control group was similar to that of the nisin group (*P *= 0.322 with *R* = 0.035) (Fig. 2E). Furthermore, nisin treatment in infected mice induced a shift in the brain microbiome, which was significantly different from that of the control and nisin group (*P* = 0.006 with *R* = 0.509 and *P* = 0.009 with *R* = 0.7, respectively) (Fig. 2E).

### Nisin attenuates the periodontal pathogen burden in the brain following oral polymicrobial infection/periodontal disease

**Fig. 3:**
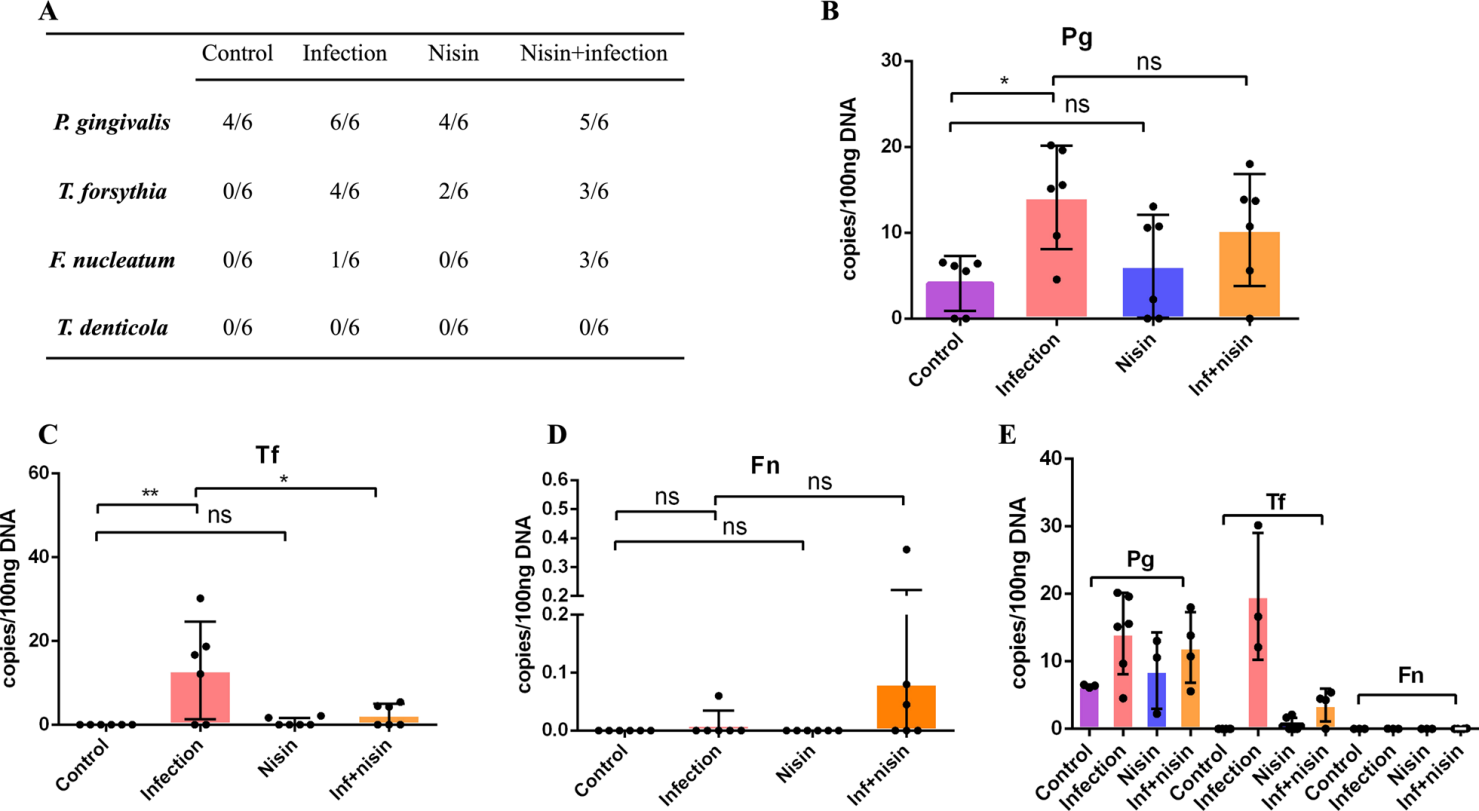
Nisin attenuates the burden of periodontal pathogens in the brain following oral polymicrobial infection. DNA was isolated and purified from the brain samples of four groups (Control, Infection, Nisin and Infection + nisin). The bacteria were quantified by standard real-time PCR using primers corresponding to 16S ribosomal RNA. **A** The table demonstrates the detection frequency (%) of periodontal pathogens in all collected brain samples. The copy numbers of each pathogen **B** *P. gingivalis*, **C ***T*.*forsythia*, and **D** *F. nucleatum*) were detected in every 100 ng DNA. *, the difference between the two groups was significant (*P* < 0.05), ns, the difference between the two groups was non-significant. **E** The copy number of each pathogen shown in aggregate for comparisons of relative levels

To further assess the presence of periodontal bacteria in the brain tissues, the number of periodontal pathogens was measured in the brain samples using RT-PCR in a manner of absolute quantification. The detection frequency of the four periodontal pathogens is shown in Fig. 3A. *P. gingivalis* could be detected in all four groups with different frequencies. *T. forsythia* was detected in the infection, nisin, and nisin + infection group, but not in the control group. *F. nucleatum* was detected in the infection, nisin + infection group, but not in the control and nisin group. The presence of *T. denticola* was not detected in any of the groups. The copy number for *P. gingivalis* in the control group was significantly lower than that in the infection group (*P* < 0.05) (Fig. 3B). Although the *P. gingivalis* copy number was lower in the infection + nisin group than in the infection group, this did not reach statistical significance (Fig. 3B). However, nisin treatment significantly reduced the *T. forsythia* copy number in the infection + nisin group compared to the infection group (*P* < 0.05) (Fig. 3C). Interestingly, the *F. nucleatum* levels were higher in the infection + nisin group than that in the infection group, but the difference was not statistically significant (Fig. 3D). When taken in aggregate, the copy numbers for *T. forsythia* were highest among all 4 pathogens in the infection group (Fig. 3E).

Using a slightly different mouse model with only a *T. denticola* infection and utilizing a sterile technique and environment, we were able to isolate whole living bacteria from the fresh brains of both control and infected mice. Using PCR validation we confirmed the presence of *F. nucleatum* in the fresh brains of the mice (Additional file 2: Fig. S2).

### Oral polymicrobial infection/periodontal disease induces neuroinflammation in the brain and nisin reverses the change

**Fig. 4:**
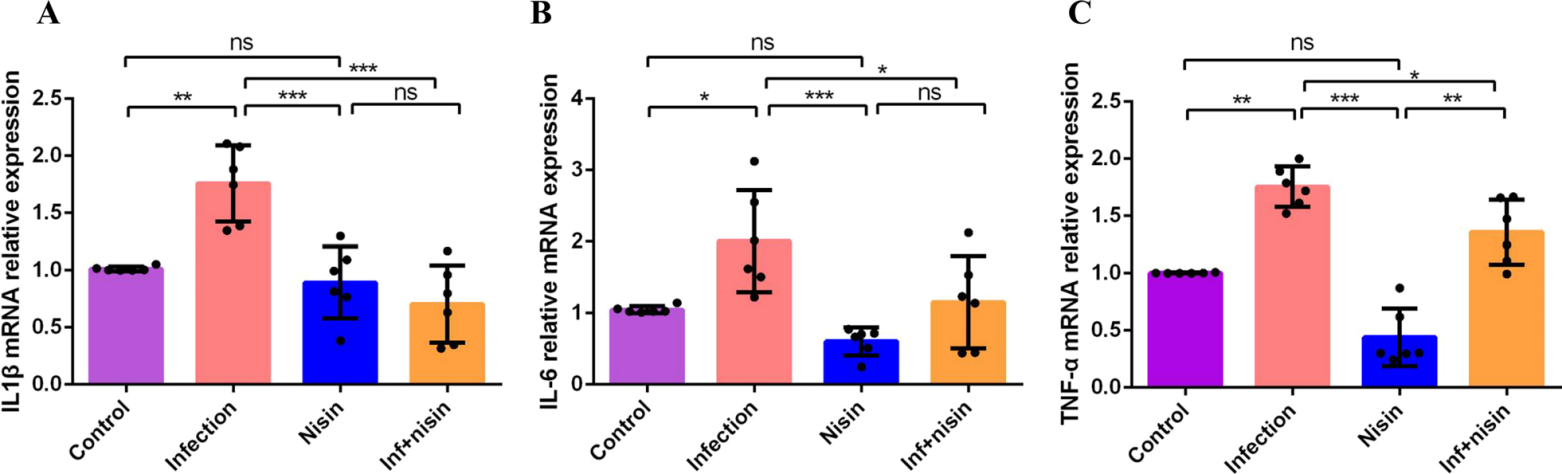
Nisin inhibits the expression of proinflammatory cytokines in the brain following oral polymicrobial infection. To evaluate the immune cytokine profiles in brain tissues, mRNA expression of IL-1β (**A**), IL-6 (**B**) and TNF-α (**C**) were measured by real-time PCR. The amount of mRNA in each reaction was normalized to GAPDH. Data are shown as means ± standard deviation from 6 mice per group. *, the difference between the two groups was significant (*P* < 0.05). **, the difference between the two groups was significant (*P* < 0.01). ***, the difference between the two groups was significant (*P* < 0.001), ns, the difference between the two groups was non-significant

To evaluate the ability of an oral polymicrobial infection to induce inflammation in the brain and the ability of nisin to relieve this neuroinflammation, we performed gene expression analysis to evaluate the immune cytokine profiles in the brain tissues. The mRNA expression of pro-inflammatory cytokines, including IL-1β, IL-6 and TNF-α were significantly higher in the infection group than that in the control and nisin group (*P* < 0.05) (Fig. 4). After nisin treatment, the expression of IL-1β and IL-6 mRNA in the infected group decreased significantly to a level similar to that in the control group and nisin group (*P* < 0.05). Nisin treatment also significantly reduced the expression of TNF-α mRNA in the infected group compared to the infection group (Fig. 4).

### Nisin abrogates the deposition of Aβ42, Tau, and phosphorylated Tau in the brain triggered by an oral polymicrobial infection/periodontal disease

**Fig. 5:**
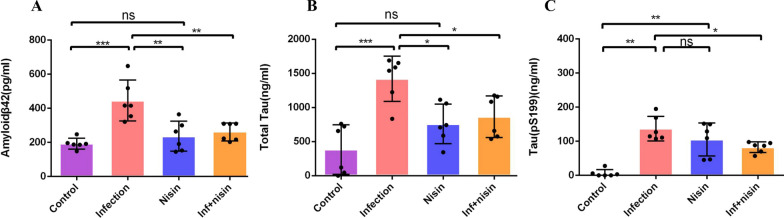
Nisin abrogates the deposition of Aβ42, Tau, and phosphorylated Tau in the brain following oral polymicrobial infection. To evaluate the effect of nisin on modulating brain pathological changes, ELISA analysis was conducted to determine the levels of Aβ42 (**A**), total Tau (**B**) and phosphorylated Tau (**C**) in brain homogenates. Data are shown as means ± standard deviation from 6 mice per group. *, the difference between the two groups was significant (*P* < 0.05). **, the difference between the two groups was significant (*P* < 0.01). ***, the difference between the two groups was significant (*P* < 0.001), ns, the difference between the two groups was non-significant

To evaluate the ability of an oral polymicrobial infection to trigger brain pathological changes and nisin’s ability to reverse these changes, enzyme-linked immunosorbent assays (ELISA) were performed to evaluate the levels of Aβ42, Tau, and phosphorylated Tau in brain tissue homogenates. As shown in Fig. 5, the concentrations of Aβ42, Tau, and Tau (pS199) were significantly higher in the infection group compared to the control group (*P* < 0.001). Nisin treatment markedly reduced the Aβ42, total Tau, and phosphorylated Tau deposition in the brain in the infection group (*P* < 0.05).

## Discussion

In the present study, we investigated the ability of an oral polymicrobial infection/periodontal disease to induce changes in the brain microbiome and key molecular biomarkers of neurodegenerative disease and the ability of nisin to mitigate these changes. We hypothesized that nisin could prevent the periodontal pathogen-mediated neurodegeneration as a direct consequence of its ability to reverse the changes in the brain microbiome, immune profiles, and pathologic protein deposition. This hypothesis was based on the observations that nisin is known to have antimicrobial effects on gram positive and gram negative oral/periodontal bacteria, antibiofilm effects, oral microbiome modulatory properties, and anti-inflammatory properties [32–34, 63, 64]. To test this hypothesis, we used BALB/cByJ mice, a common mouse strain, to establish periodontal disease by oral infection with four key periodontal pathogens. Different mouse models of periodontal infection have been used to investigate the role of periodontal disease in the pathogenesis of AD [24, 28, 42–45]. Most of the models used *P. gingivalis* or its lipopolysaccharide as the only pathogenic bacteria or virulence factor. However, infection with a single periodontal pathogen does not recapitulate the polymicrobial nature of periodontal disease. Studies have indicated that the host immune responses to a polymicrobial infection are different from responses to a mono-infection [46, 47]. Our previous studies demonstrated that an oral polymicrobial infection in BALB/cByJ mice led to significant alveolar bone loss, a heightened antibody response to the periodontal pathogens, and an elevated cytokine immune response, indicating that this model is a representative and useful model of periodontal disease [34, 35, 41]. Therefore, this polymicrobial mouse model of periodontal disease is an improvement over mono-infection models, and thus useful for evaluating the relationship between periodontal disease and systemic diseases and the effect of treatment modalities.

Over the last decade, a growing body of scientific evidence has demonstrated that alterations in the oral microbiota play an important role in the initiation and progression of AD. Oral microbiota could be transported to the brain through the blood stream in patients with periodontitis [48]. Among the oral microorganisms, *P. gingivalis*, *T. forsythia, F. nucleatum* and *T. denticola* have been implicated in the development of AD [21, 22, 49]. *P. gingivalis* is the most documented periodontal pathogen in AD-related studies. The presence of *P. gingivalis* and its virulence factors, including lipopolysaccharide and gingipains, were confirmed in this context in both human and animal studies [24, 28, 50]. In the present study, *P. gingivalis* DNA was detected in all six mice in the infection group, which further confirmed the translocation of *P. gingivalis* components from oral cavity to the brain. However, *P. gingivalis* was also detected in four of the six control mice at the conclusion of the study. There could be several reasons for this observation. We and others have previously shown that mice carry *P. gingivalis* in the oral cavity, thus this may be the source of the brain *P. gingivalis* signal in the control mice, despite our initial antimicrobial washout period [34]*.* Another reason for this result may be that the mice were not housed in a gnotobiotic or specific pathogen free (SPF) grade facility, and thus there may have been potential environmental contamination; although the animals had an initial antimicrobial wash out period. However, the quantitative PCR results showed that the number of *P. gingivalis* copies in the infection group were significantly higher than that in the control group, indicating that oral infection did result in an increased load of *P. gingivalis* DNA in the brain tissues. In contrast, *T. denticola* was not detected in the brain samples in our study, which is inconsistent with former studies [51, 52]. Miklossy first proposed that some spirochetes derived from the oral cavity were associated with AD [49]. Riviere et al. further demonstrated that several types of oral *Treponema* species, including *T. denticola* have been found in AD brain samples [21]. In our previous study, we found that it was relatively hard for *T. denticola* to colonize the oral cavity compared with the other three bacteria [35]. At the end of the experimental period, only two of the six mice in the infection group had detectable *T. denticola*, and the proportion of *T. denticola* among total bacteria was very low. Therefore, we surmise that the limited number of *T. denticola* that colonized the oral cavity were not sufficient to disseminate and infect the brain. Although the IgG levels to *T. forsythia* and *F. nucleatum* are elevated in AD patients [53], these two periodontal pathogens have not been detected in brain tissues. Our study, for the first time, demonstrates the possibility that *T. forsythia* and *F. nucleatum* DNA can be transported from the oral cavity to the brain. The *T. forsythia* bacterial load in the brain of infected mice was higher than that of *P. gingivalis*, suggesting that *T. forsythia* may possess specific virulence factors that promote its translocation to the brain. Therefore, *T. forsythia* may be another important periodontal pathogen that links periodontal infection with AD pathology. *F. nucleatum* was also detected in the brain of the infected mice but at extremely low levels compared to that of *P. gingivalis* and *T. forsythia*. Therefore, the role of *F. nucleatum* in AD pathology may be similar to that of its role in dental plaque formation [54], that is, it may act as a "microbial bridge" to communicate with other bacteria.

Importantly, in a slightly different oral infection mouse model, we found the presence of whole live oral bacteria present in fresh mouse brains from both control and infected mice (Additional file 2: Figure S2). Furthermore, we used PCR validation to confirm that one of the live bacteria present in the brains, included a periodontal pathogen, namely, *F. nucleatum*. This speaks to the idea of a natural living microbial presence in the brains of mice and supports the concept of a natural brain microbiome.

In addition to individual bacteria, dysbiosis can also contribute to AD pathogenesis. Gut and oral dysbiosis have been implicated in AD development and progression [42, 55–57]. However, there is no direct evidence supporting a correlation between alterations in the microbial profile of the oral cavity and AD brain. Using 16s rRNA sequencing, we found that the microbiome composition of the brain of the periodontally infected mice was significantly different from that of the control mice. The relative abundance of *Firmicutes* and *Proteobacteria* was significantly higher in the brain after polymicrobial–periodontal infection, and the proportions of *Actinobacteria*, *Bacteroidetes* and *Cyanobacteria* were much lower. These altered dominant phyla may not possess periodontal pathogenicity, and may not originate from the oral cavity, but these changes could be the result of oral microbial alterations. Therefore, periodontal infection may not only lead to the transfer of periodontal pathogens from oral cavity to brain, but more importantly, it may also contribute to changes in the microbiome composition of the brain. The potential for a periodontal infection to promote an increase in brain pro-inflammatory cytokines, including IL-1β, IL-6 and TNF-α, which ultimately promote the production of Aβ and hyperphosphorylation of tau, resulting in neurodegeneration and AD has been postulated [58]. Therefore, restoring the composition of the oral microbiome could be a helpful approach and possible therapeutic intervention strategy for AD. Recently, our group demonstrated that nisin can prevent and disrupt oral biofilms, decrease the amount of oral pathogens within oral biofilms, and return the diversity and composition of diseases-associated oral biofilms back to control levels, demonstrating that nisin can modulate pathogenic oral biofilms towards health in vitro [33]. In this study, the effect of nisin treatment on the translocation of periodontal pathogens to the brain was also evaluated. We found that although treatment with the nisin bacteriocin did not significantly change the bacterial load of *P. gingivalis* and *F. nucleatum*, it did reduce the number of *T. forsythia* in the brain of infected mice, suggesting that the antibacterial effect of nisin was varied among different bacteria in this community setting with different modulating mechanisms. Our study further discovered that nisin treatment resulted in significant alterations in the brain microbiome. Nisin treatment dramatically increased the relative abundance of *Proteobacteria* but decreased the proportions of *Firmicutes*, *Actinobacteria*, *Bacteroidetes* and *Fusobacteria*. A study on the bacterial diversity of subgingival plaque showed that the predominant species in the subgingival plaque included *Proteobacteria, Actinobacteria*, *Bacteroidetes*, *Firmicutes*, *Fusobacteria* and *Spirochetes*, and the latter five phyla were closely related to periodontal disease pathogenesis [59]. The results of our study indicate that the dominant bacterial species of the brain bacterial community in periodontally infected mice is similar to that of the oral microbiota. In addition, nisin reduced the relative abundance of several species in the brain, including *Firmicutes*, *Actinobacteria*, *Bacteroidetes* and *Fusobacteria,* which may be the consequence of nisin’s ability to alter the oral microbial community (Additional file 1: Fig. S1; [34, 41]).

Importantly, we found that nisin treatment downregulates the expression of pro-inflammatory cytokines and reduces the deposition of Aβ42 and phosphorylated tau proteins in the brain of periodontally infected mice. The “inflammation hypothesis of AD” proposed by Krstic and Knuesel [60] is one of most important hypotheses in the pathogenesis of AD. Neuroinflammation is an inflammatory response to injury or infection in the central nervous system (CNS). It is well-known that microglia and astrocytes may be activated during this process, which produce excessive pro-inflammatory cytokines, especially IL-1β, IL-6, TNF-α, and additional Aβ formation [61, 62]. Therefore, our study indicates that nisin relieves inflammation in the brain, thereby reducing the production of Aβ42 and phosphorylated tau protein, which are two characteristic pathological changes of AD brains.

Although nisin is known to modulate inflammation and the host immune response [63, 64], at present, there is limited research on the regulatory mechanism of nisin on neuroinflammation in microglia/astrocytes. Zainodini et al. reported that nisin induces cytotoxicity and apoptosis in a human asterocytoma cell line (SW1088) [65]. Apoptosis is considered a form of physiological death that does not initiate an inflammatory response [66]. In contrast, recent research has reported that the apoptotic metabolite secretome induces specific gene programs in healthy neighboring cells, including suppression of inflammation [67]. Based on these reports, we surmise that nisin may regulate neuroinflammation in microglia/astrocytes through apoptosis-related mechanisms, and related studies deserve further exploration.

Growing evidence has demonstrated that antimicrobial peptides (AMPs) are capable of penetrating cell membranes and crossing the blood brain barrier [68, 69]. Importantly, nisin is known to preferentially intercalate into phospholipid membranes and it forms pores in lipid membranes [70–74]. The blood–brain barrier opening (BBBO) mechanism utilizes reversible disruption of cell–cell junctions between brain microvascular endothelial cells to enable transient entry of molecules into the brain. Linville RM et al. found that a Melittin peptide and other membrane active variants transiently increase paracellular permeability via disruption of cell–cell junctions that result in transient focal leaks; this may be one of the mechanisms by which antimicrobial peptides enter the blood–brain barrier [75]. Nisin, as an antimicrobial peptide, may enter the blood–brain barrier through a similar mechanism.

Thus, mechanistically, nisin may exert multiple effects in this periodontal disease–AD model as a result of its ability to interact with/modulate cell membranes; this is the basis for its antimicrobial properties (binding lipid II on bacterial cell membranes and forming pores), and it may also be gaining entry into the brain on this basis (since nisin treatment alone changes the brain microbiome composition; Fig. 1). Furthermore, nisin also modulates host inflammation. Therefore, nisin may be impacting oral pathogen levels/oral microbiome levels directly, resulting in lower pathogenic bacteria/bacterial DNA potentially transferred to the brain through the blood stream. Although, nisin may also be crossing the blood–brain barrier to modulate neuroinflammation–stemming from brain microbiome changes.

## Limitations

The study findings are based on animal studies and, therefore, are not directly generalizable to the human condition. In addition, the animal model used is a mouse model of periodontal disease, and therefore, the results are relevant and limited to this condition. Although a sample of size of 6 mice per group may seem limited, it was selected based on prior studies with this mouse of model of periodontal disease, which revealed significant differences in oral parameters and microbiome changes and similarly here revealed significant differences in the outcome measures [34, 35, 41].

## Conclusions

Our study indicates that an oral polymicrobial–periodontal infection/periodontal disease can extend the effects of periodontal pathogens, including *P. gingivalis*, *T. forsythia* and *F. nucleatum* from the oral cavity to the brain, and thereby induce a shift in the oral–brain–microbiome axis, produce neuroinflammation via production of proinflammatory cytokines, and produce Aβ and phosphorylated tau proteins in the brains of mice. Importantly, we provide evidence that nisin can effectively abrogate these changes by altering the composition of the brain microbiome after periodontal infection, mitigating inflammatory cytokine release, and reducing the Aβ load and hyperphosphorylation of tau; demonstrating a potential role for nisin in the prevention and treatment of AD.

### Supplementary Information

**Additional file 1: Figure S1. **Analysis of the microbial abundance by 16s rRNA sequencing show that nisin shifts the oral microbiome back towards healthy control levels following infection. The groups included Control, Infection, Nisin, Infection + Nisin. Differential abundance analysis for bacteria at the phylum level. Data previously highlighted in Kuraj et al., 2023 [41].

**Additional file 2: Figure S2. **Live bacteria sampled from the brain and PCR confirmation of *F. nucleatum*. In a *T. denticola* oral infection mouse model, brain swabs that were collected in a sterile environment with a sterile technique revealed bacterial growth (A). This bacterial growth was sampled and subsequently plated (B). Then, multiple random colonies from the plates were amplified/checked by RT-PCR with primers specific for periodontal pathogens (*T. denticola, P. gingivalis, F. nucleatum, T. forsythia)*. The bar graph shows the Ct values for *F. nucleatum* detected from control and infected brain samples.

## Data Availability

The data and code are available at https://figshare.com/articles/dataset/raw_data_Zhao/22586137.
