# Supplementary figures and images for: Nisin a probiotic bacteriocin mitigates brain microbiome dysbiosis and Alzheimer’s disease-like neuroinflammation triggered by periodontal disease
Source: J Neuroinflammation. 2023 Oct 6;20:228. doi: 10.1186/s12974-023-02915-6 (PMC10557354; doi:10.1186/s12974-023-02915-6)

# Oral microbiome

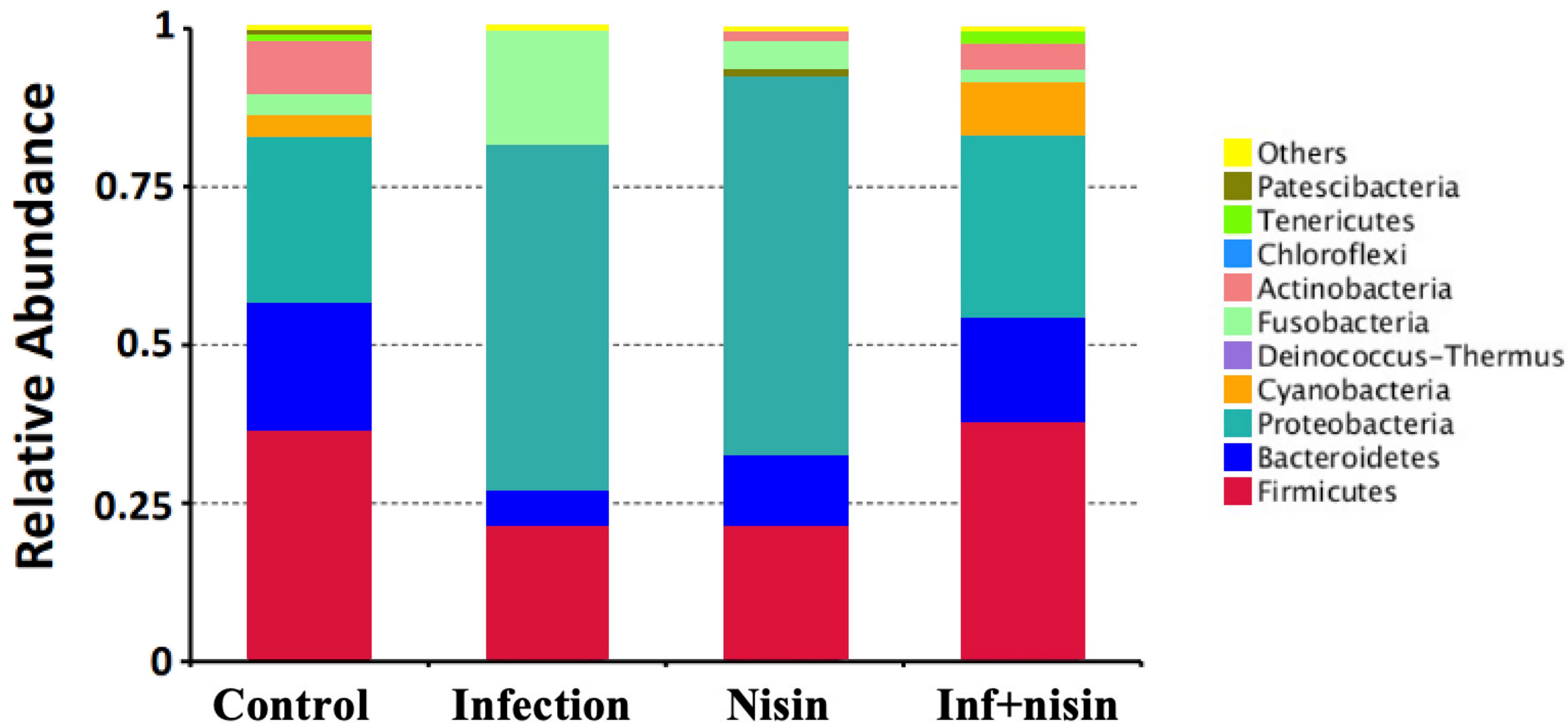

Supplement: Supplementary file 1 — Additional file 1: Figure S1. Analysis of the microbial abundance by 16s rRNA sequencing show that nisin shifts the oral microbiome back towards healthy control levels following infection. The groups included Control, Infection, Nisin, Infection + Nisin. Differential abundance analysis for bacteria at the phylum level. Data previously highlighted in Kuraj et al., 2023 [41]. [file 12974_2023_2915_MOESM1_ESM.pdf]

**A**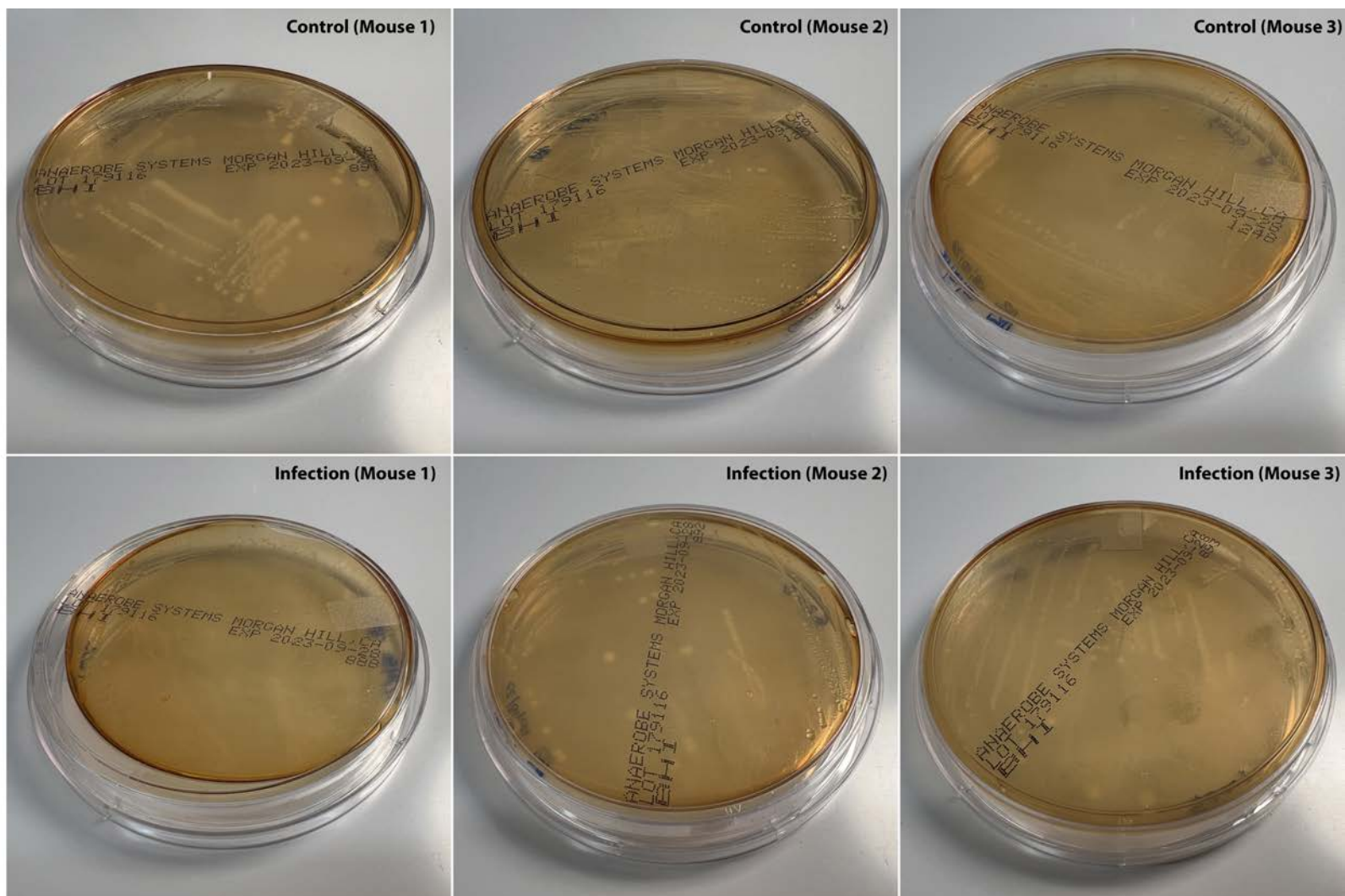**B**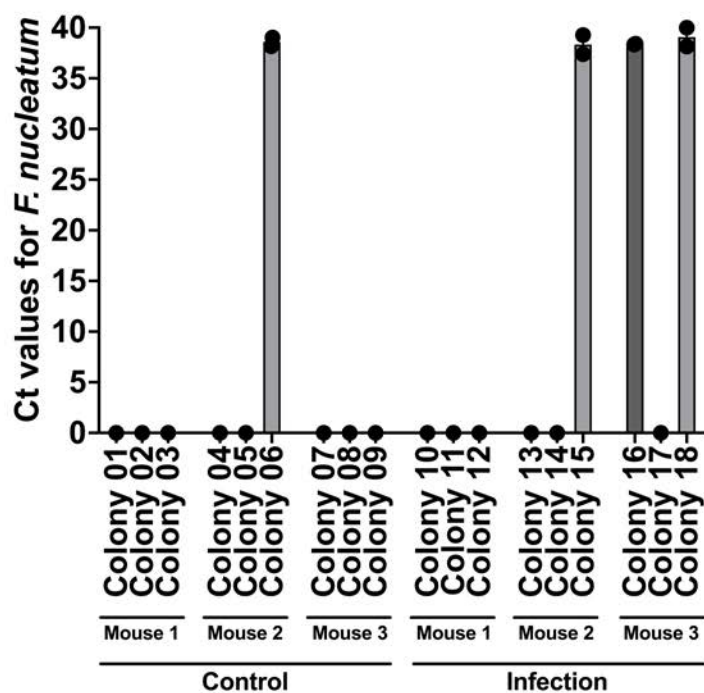

Supplement: Supplementary file 2 — Additional file 2: Figure S2. Live bacteria sampled from the brain and PCR confirmation of F. nucleatum. In a T. denticola oral infection mouse model, brain swabs that were collected in a sterile environment with a sterile technique revealed bacterial growth (A). This bacterial growth was sampled and subsequently plated (B). Then, multiple random colonies from the plates were amplified/checked by RT-PCR with primers specific for periodontal pathogens (T. denticola, P. gingivalis, F. nucleatum, T. forsythia). The bar graph shows the Ct values for F. nucleatum detected from control and infected brain samples. [file 12974_2023_2915_MOESM2_ESM.pdf]
